# Comparison of Closed-Ended, Open-Ended, and Perceived Informed Consent Comprehension Measures for a Mock HIV Prevention Trial among Women in Tanzania

**DOI:** 10.1371/journal.pone.0105720

**Published:** 2014-08-26

**Authors:** Kathleen M. MacQueen, Mario Chen, Catalina Ramirez, Soori E. A. Nnko, Kelly M. Earp

**Affiliations:** 1 Global Health, Population and Nutrition, FHI 360, Durham, North Carolina, United States of America; 2 Sexual and Reproductive Health, National Institute for Medical Research (NIMR), Mwanza, Tanzania; UCL Institute of Child Health, University College London, United Kingdom

## Abstract

Verifying participant comprehension continues to be a difficult ethical and regulatory challenge for clinical research. An increasing number of articles assessing methods to improve comprehension have been published, but they use a wide range of outcome measures including open-ended, closed-ended, and self-perceived measures of comprehension. Systematic comparisons of different measures have rarely been reported. This study evaluated the likely direction of bias observed when using open-ended, closed-ended, and perceived ease of comprehension measures among women administered a mock informed consent process in Mwanza, Tanzania. Participants were randomized to either a closed-ended or an open-ended assessment of comprehension, administered the consent process for a hypothetical HIV prevention trial in Kiswahili, and then administered a comprehension assessment, per their randomization. They were then asked how easy or hard it was to understand each of the informed consent components measured in the comprehension assessment. Women in the closed-ended arm had significantly higher overall comprehension scores than in the open-ended arm. Perceived scores were significantly higher when compared to both open-ended and close-ended scores within arms but were similar between arms. Findings highlight the importance of comprehension assessments in complex clinical trials that go beyond asking participants if they understand or have any questions. They also indicate the need for continued exploration of objective measures of comprehension in international clinical research settings, so that points in need of clarification can be efficiently and effectively identified and addressed. Such measures would reduce burdens on both staff and participants that result from well-intentioned but potentially unnecessary time spent explaining in unwarranted detail things already understood.

## Introduction

Multiple studies have documented the challenges study participants face in comprehending the content of informed consent documents for clinical trials. [1]–[5]. For example, participant comprehension may be compromised by a lack of education [6]–[8], difficulties in translating scientific terms [9], [10], and difficulties with processing large amounts of information in a time-limited setting [1], [11].

Prevention trials bring additional challenges with regard to comprehension of risks and benefits because they are implemented with healthy people who are determined to be at risk for a disease, but whether a specific individual will in fact experience that disease in the absence of preventive measures is not predictable. Thus the research risks are borne by the individuals in the trial but the benefits mainly accrue for the group as a whole; there may be no direct benefit at the individual level. These challenges are further complicated for biomedical HIV prevention trials by the risks related to stigma, discrimination, and other social harms that have historically traveled with the epidemic [12]. Concerns about the potential for exploitation in the context of HIV prevention trials have resulted in close scrutiny of consent processes to insure participants are fully informed and comprehending of the risks involved [13], [14].

Verifying participant comprehension has proven to be a difficult ethical and regulatory challenge for clinical research in general. This is true even under optimal conditions, such as high literacy and familiarity with advanced Western medical practices; it may be even greater in non-Western, low-literacy, resource-poor settings. Efforts to address this problem have led to the development of various comprehension assessment methodologies, particularly in the context of biomedical HIV prevention trials. Close-ended approaches have been used in both industrialized countries (e.g.: United States) and, to a limited extent, in resource-poor settings including Thailand, Haiti, and South Africa [1], [15]–[18]. More recently, open-ended methodologies for assessing comprehension of informed consent have been developed, such as vignettes and narratives [19].

Identifying the best method for improving participant comprehension is closely linked to the issue of how comprehension is assessed. A study conducted among people screening for HIV vaccine trials in South Africa suggested that qualitative approaches such as open-ended questions and discussion of vignettes may be more effective at pin-pointing areas of poor comprehension than are checklists and closed-ended responses [19]. Such qualitative approaches are intensive, however, and may result in extended conversation on topics where comprehension is fine in order to identify selected topics where it is inadequate. This results in added burden for both staff and participants. Limited research has been conducted on the burden aspect of informed consent. At least one study has demonstrated discrepancies between participants' subjective understandings of informed consent elements and what they desire to know with regard to those elements, suggesting that informed consent could be improved by placing more emphasis on elements where participants perceive they are under-informed [20]. However, an earlier study indicated that perceived comprehension may in fact overestimate what research participants understand [15].

As part of a larger study exploring verbal and nonverbal indicators of informed consent comprehension, we sought to verify the likely direction of bias observed when using open-ended and closed-ended comprehension assessments. In addition, we compared the measures of assessment with participants' self-report of the perceived ease of comprehension for each of the specific elements of informed consent.

## Methods

### Ethics Statement

The study was approved by the Tanzanian National Health Research Ethics Review Sub-Committee (NatREC) and the Protection of Human Subjects Committee at FHI 360, USA. Participants provided written informed consent to participate in the study and the process was approved by the ethics committees.

### Research Setting

The study was implemented in Mwanza, a region of Tanzania with a population of 2.7 million [21]. Field implementation was led by the National Institute of Medical Research (NIMR), which had ongoing HIV prevention trials and experienced informed consent counselors trained in accordance with Good Clinical Practice (GCP) standards. HIV prevalence among adults 15–49 years old was estimated at 5.8% in Tanzania in 2011 [22].

Prior to study implementation the NIMR study team met with key stakeholders including representatives from the local and national government, non-governmental organizations working in the area, local community gatekeepers and leaders, owners or managers/operators of recreational facilities such as bars, pombe shops (local brew facilities), guest houses, hotels, *mamalishe* stalls/restaurants (food vendors), and community based health organizations. The meetings provided opportunities to obtain necessary approvals, garner community support for the study, and aid in identification of facilities appropriate for participant recruitment. Additionally, owners, managers, or supervisors of the identified facilities were invited for introductory meetings to provide them with study information, explain recruitment procedures and obtain the owner's, manager's, or supervisor's support to recruit at their facility. These were procedures typically used by NIMR for various field research prior to implementing research in the country.

### Participants

To be eligible for recruitment, participants needed to be women between the ages of 18 and 35, currently residing in Magu or Misungwi Districts, and not intending to move outside the study area in the next month. Additional eligibility criteria mirrored those commonly defined for sexual and reproductive health clinical trials conducted by NIMR in the Mwanza area: at least one vaginal sex act in the past 14 days or having more than one sexual partner in the past 30 days. Women who reported ever having participated in a clinical research study were excluded. Unlike most HIV prevention trials of the time, where eligibility criteria would typically exclude those who are HIV-infected, we did not screen for HIV status or use HIV testing to determine eligibility.

Participants were recruited at venues contacted during the community engagement activities and identified as potential recruitment sites for future biomedical HIV prevention trials. During recruitment, study staff spoke with a variety of employees and clients, including men, in an effort to protect participant confidentiality and avoid stigmatizing women. General information about the study and the types of research activities included was provided to each potential participant. Screening was conducted at venues and eligible women expressing interest in participation were given an appointment time to complete the study activities.

### Research Design

All participants completed informed consent at enrollment; the informed consent process explained that participants would be administered a mock informed consent process for a hypothetical clinical trial. After completing all study procedures, participants were debriefed to ensure that they understood that the clinical trial described in the mock informed consent process was not being implemented and that they had not consented to be in such a trial. The hypothetical clinical trial design was a randomized, blinded, placebo-controlled trial to assess the effectiveness of a daily oral pill to prevent acquisition of HIV, an intervention referred to as pre-exposure prophylaxis or PrEP. At the time of the study, several such PrEP trials were underway globally [23].

At enrollment participants were randomized to one of two informed consent conditions: IC-C which included a closed-ended assessment of comprehension or IC-O which included an open-ended assessment. Group assignments were concealed in sequentially numbered, sealed opaque envelopes. Staff opened the envelopes sequentially after a participant was enrolled to determine which condition that participant was randomized to receive.

Following randomization participants were administered a short sociodemographic face-to-face interview. All participants were then administered the same mock informed consent process in Kiswahili for a hypothetical trial called the HIV-PrEP Clinical Trial Study. The level of detail included in the informed consent form for the mock process was derived from several informed consent processes either previously used or currently being used in PrEP trials. The complete English version of the consent form was 4705 words; it was translated into Kiswahili using standard NIMR procedures, which included several rounds of back-translation. During the mock process a trained research assistant read the informed consent form to the participant, per Good Clinical Practice (GCP) requirements; the participant could follow along with her own copy if she could read. An impartial witness was not provided for illiterate participants (normally this is a CGP requirement). The mock informed consent form explained that the purpose of the trial was to find out if a drug called PrevVI was safe and could reduce the chance that women get HIV from sex, that PrevVI was a pill that needed to be taken once a day, that it was made from two different types of antiretroviral drugs (ARVs), and that ARVs are used to treat people with HIV. The design of the hypothetical study—a double-blind randomized placebo-controlled trial—was explained, along with procedures, number and frequency of study visits, potential risks and benefits, what would happen if she became HIV-infected or pregnant during the trial, collection of blood samples and storage for future use, compensation, and rights as a participant. The potential risks were modelled after those associated with a combination pill that includes tenofovir disoproxil fumarate (TDF) and emtricitabine (FTC) and that has been evaluated in many PrEP studies [23].

Participants were then administered the comprehension assessment, either IC-C or IC-O per their randomization. After the comprehension assessment was concluded, participants were asked to complete a self-perception comprehension assessment (IC-SP), where each woman was asked how easy or hard it was to understand the key concepts evaluated by the IC-C and IC-O comprehension assessment tools.

In a typical biomedical HIV prevention trial, participants would be asked to sign the informed consent form once they had demonstrated adequate comprehension. In this study, women were not asked to sign the mock informed consent form because we did not want to cause any confusion about the intent of the study. Rather, they were asked how likely or willing they would be to participate if eligible and how willing they thought other women in Mwanza would be to participate if those women were eligible. In alignment with the construct definition of informed consent comprehension developed by Buccini and colleagues [24] this hypothetically allowed a proxy assessment of the relationship between comprehension and willingness to participate, and thus was intended to simulate the decision-making point in the consent process.

### Measurement

Sociodemographic information collected during the face-to-face interview included age (based on year born), highest level of school attended, religion, current marital status, and whether the participant personally knew someone who had or had died from the AIDS virus. Literacy was assessed by asking the participant to read a simple sentence in Kiswahili: “*Kilimo ni kazi ngumu*” (“Farming is hard work”); the sentence is one that has been used for this purpose in the Demographic Health Survey in Tanzania [25].

The comprehension assessment tools were derived from models used in successfully completed trials with multiple levels of international ethics and regulatory review. The IC-C assessment was adapted from a true/false comprehension assessment tool developed initially as part of preparatory research for the VAXGEN Phase III HIV vaccine trial conducted in Bangkok [16], [26]; this was modified for use with the hypothetical PrEP trial consent form. The IC-O assessment was similarly adapted from an open-ended checklist tool used in the HPTN 035 Phase II microbicide trial conducted at multiple sites in Africa and the U.S [27]. The comprehension points included in the IC-O checklist and the self-perception comprehension assessment (IC-SP) corresponded to the comprehension points included in the IC-C assessment, thus allowing a direct comparison across all of the measures.

The assessment was comprehensive and detailed, including seven components with multiple specific items totaling 39 comprehension points. The components reflect required elements of informed consent for clinical research (e.g., purpose of the research, possible risks, confidentiality) while the points reflect the specific content included in the mock informed consent to address those required elements. For each comprehension point from the IC-C, IC-O, and IC-SP, the following measures were generated (Table 1):

**Table 1:** Informed consent comprehension components, points, and associated measures.

Comprehension component	Comprehension point as worded for perceived ease of understanding (IC-SP)	Closed-ended comprehension Questions (IC-C); True/False response	Open-ended comprehension questions (IC-O)
***Purpose of the research; maximum score = 6***	There are different pills, one with the PrevVI drug and one without (a placebo)	There are different pills, one with the PrevVI drug and one without (a placebo) (T)	How is the drug being tested among women in the study? *Probe: Anything else?*
	Not everyone receives a drug in the pill	Everyone receives a drug in the pill (F)	Two groups of women will take a pill in this study. How is it decided who will receive the pill with a drug, or the pill without a drug? *Probe: Who will know whether the woman gets the pill with the drug or without the drug?*
	The study is experimental	The study is experimental (T)	
	No one knows who receives which pill	The study staff will know who receives which pill (F)	
	The study is testing a drug to see if it can prevent HIV infection	The study is testing a drug to see if it can prevent HIV infection (T)	Why are we doing this study? *Probe: Anything else?*
	The study is testing to see if the drug is safe for prevention	The study is testing to see if the drug is safe for prevention (T)	
***Study procedures; maximum score = 9***	If you were in the study, you will need to take a study pill once a day	Participation in the study includes taking the study pill once a day (T)	What are women being asked to do in this study? *Probe: Anything else?*
	If you were in the study, you will need to use condoms for every sex act	Participation in the study includes using condoms some of the time only (F)	
	If you were in the study, you will need to come for clinic visits about every 4 weeks for 18 months	Participation in the study includes coming for clinic visits about every 4 weeks for 18 months (T)	
	If you were in the study, you will need to bring bottles of pills with any remaining pills in it to every clinic visit	Participation in the study does not include bringing bottles of pills with any remaining pills in it to every clinic visit (F)	
	If you were in the study, you will have physical and pelvic exams	Participation in the study includes having physical and pelvic exams (T)	
	If you were in the study, your blood will be drawn at each clinic visit	Participation in the study includes having blood drawn at the first and last clinic visit only (F)	
	If you were in the study, you will be asked questions about your behavior and health	Women in this study will be asked questions about their behaviour and health (T)	
	If you were in the study, you will need to take study approved contraception for the duration of your study participation	Participation in the study includes taking study approved contraception for the duration of the study (T)	
	If you were in the study, you will need to provide contact information and update study staff if it changes	Participation in the study includes providing contact information and updating study staff if it changes (T)	
***Possible risks; maximum score = 8***	If you were in the study, you may experience minor side effect from study pill, such as nausea, vomiting, diarrhea, gas or bloating of the stomach, rash, and/or headache	Women in this study will definitely experience minor side effects from study pill, such as nausea, vomiting, diarrhoea, gas or bloating of the stomach, rash, and/or headache, as a result of study participation (F)	What are the risks of being in this study? *Probe: Please list any minor risks. (Be able to name at least one side effect)*
	If you were in the study, you may experience rare but serious side effect from the study pill, such as problems related to your liver, kidney, bone density, and/or allergy	Women in this study are at risk of experiencing rare but serious side effects from the study pill, such as problems related to your liver, kidney, bone density, and/or allergy, as a result of study participation (T)	*Probe: Please list any major risks.* (*Be able to name resistance and at least one rare but serious side effect)*
	If you were in the study, you may develop resistance to PrevVI or some other types of ARVs if you become HIV positive	If a woman in this study becomes HIV positive as a result of study participation, she may be at risk of developing resistance to PrevVI or other types of ARVs (T)	
	If you were in the study, you may experience problems with other people if they tell or others find out that you have taken part in this trial	Women in this study are not at risk of experiencing problems with other people if they tell others, or others find out, that they have taken part in this trial (F)	*Probe: Please list any non-medical risks.*
	If you were in the study, you may feel discomfort, dizziness, bruising, swelling, or infection from blood draws	Women in this study may feel discomfort, dizziness, bruising, swelling, or infection from blood draws (T)	*Probe: Anything else*?
	If you were in the study, you may feel discomfort during physical and pelvic exams	Women in this study may feel discomfort during physical and pelvic exams (T)	
	If you were in the study, you may become embarrassed, worried, or anxious when asked questions about your sexual behavior or when receiving IV counseling	Women in this study may become embarrassed, worried, or anxious when asked questions about their sexual behaviour or when receiving HIV counseling (T)	
	If you were in the study, you may feel anger or distress if you learned that you are infected with HIV or other infections that are passed by sex	Women in this study may feel anger or distress if they learned that they are infected with HIV or other infections that are passed by sex (T)	
***Possible benefits; maximum score = 8***	If you were in the study, you may not have any direct benefit from being in this study	Women in this study may not have any direct benefit from being in this study (T)	What are the benefits to women in this study? *Probe: How may you benefit while you are taking part in the study?*
	If you were in the study, you or others may benefit in the future from information learned in this study	Women in this study or others may benefit in the future from information learned in this study (T)	*Probe: How may you or others benefits after the study has been completed?*
	If you were in the study, you will be given free condoms in this study	Women in this study will be given free condoms in this study (T)	*Probe: Anything else?*
	If you were in the study, you will be given free treatment of infections passed through sex during your participation in the study	Women in this study will be given free treatment of infections passed through sex during and after their participation in the study (F)	
	If you were in the study, you will be given free physical and pelvic exams during your participation in the study	Women in this study will be given free physical and pelvic exams during their participation in the study (T)	
	If you were in the study, you will be given free study approved contraception for the duration of the study	Women in this study will be given free study approved contraception for the duration of the study (T)	
	If you were in the study, you will be given general health screening and advice	Women in this study will be given general health screening and advice (T)	
	If you were in the study, you will receive HIV testing in this study	Women in this study will need to pay for each HIV testing in this study (F)	
***Confidentiality; maximum score = 3***	If you were in the study, only people working on the study will have access to your information	Only people working on the study will have access to your information (T)	How will information be protected for women in this study?
	If you were in the study, your name will not appear on study records	Names of women in this study will appear on study records (F)	*Probe: Anything else?*
	If you were in the study, contacts for missed clinic visits will be discreet	Contact for missed clinic visits will be discreet (T)	Who may come to know of a woman's missed clinic visit? *Probe: How would you describe the way study staff will contact a woman if they miss a clinic visit?*
***Contacts for questions about research and rights; maximum score = 2***	If you were in the study, you should contact the Principal Investigator if you have questions about the research study	If women in this study have questions about the research study, they can contact the Principal Investigator (T)	What should women do if they have any questions or concerns about the study?
	If you were in the study, you should contact the ethics committee representative if you have questions about your rights as a participant of the study	If women in this study have questions or concerns about their rights as a participant of the study, they can contact ethics committee representative (T)	
***Voluntariness; maximum score = 3***	If you were in the study, you are free to make your own decisions about joining the study	Women in this study are free to make their own decisions about joining the study (T)	Who makes the decision for a woman to join the study?
	There is no effect on people's access to care or services whether or not you decide to join the study	Access to care or services will be affected by whether or not a woman decides to join the study (F)	How will her health services be affected by her decision to join the study or not?
	If you were in the study, you can leave the study at any time	Women who choose to participate can only leave the study after they have completed all the study activities (F)	When can a woman leave the study?

- In the **closed-ended arm (IC-C)** of the study, participants were read a statement corresponding to each comprehension point and asked whether the statement was true or false. A dichotomous variable was generated that indicated whether they responded correctly (1) or incorrectly (0) for that point of comprehension.
- In the **open-ended arm** **(IC-O)** of the study, participants were asked to describe each of the seven components; the interviewer was trained to also use standardized open-ended probes. For each point of comprehension associated with a component, the interviewer noted whether that point was described by the participant. A dichotomous variable was generated that indicated whether the participant mentioned (1) or did not mention (0) an item for each point of comprehension as determined by the counselor/interviewer.
- Following the comprehension assessment, all participants were read an accurate statement corresponding to each comprehension point and asked their perception **(IC-SP)** regarding how easy or difficult it was to understand that point. A dichotomous variable was generated that indicated whether the participant perceived each point of comprehension as very or somewhat easy (1) or very or somewhat difficult (0) to understand.

To assess willingness to participate in the hypothetical trial, women were first asked if they thought they would be eligible to participate. If they said yes, they were then asked “How likely are you to participate in this study?” If they said they did not think they would be eligible, they were asked “If you were eligible, how willing would you be to participate in this study?” All women were then asked “How willing would other women from Mwanza region be to join this pretend study if they were eligible?”

### Data Analysis

Frequencies and percentages for discrete variables, and means, standard deviations, minima, maxima, and median for continuous variables were used to summarize sociodemographic characteristics of the sample population and assess comparability across arms.

Overall comprehension scores were calculated by summing the dichotomous variables for responses to each measurement tool (IC-C, IC-O, and IC-SP). Component comprehension scores were similarly calculated. Within each study arm we calculated means and standard deviations overall and by component for the associated comprehension tool (IC-C or IC-O) as well as for the IC-SP responses for the participants in each arm. We used paired t-tests and 95% confidence intervals (CI) for the mean score differences to assess similarity in comprehension and perceived ease of comprehension within each arm. We used t-tests for independent samples and 95% CIs to assess differences between arms in comprehension assessment and perceived ease respectively.

Statistical analyses were performed using SAS software, version 9.3. Given the small sample size, and the exploratory nature of the study, the results from these analyses are intended as an initial assessment of the comprehension assessment tools with the benefits of a randomized study to strengthen comparability between arms.

## Results

In total 80 women were enrolled, with 40 per randomization arm. Women were fairly evenly distributed across age groups; they tended to have no more than primary education, and to be literate, married or cohabitating, and Christian. Most knew somebody who had participated in a sexual or reproductive health-related clinical trial. Most also knew somebody who had died from AIDS. No major differences were observed between arms, however women in the IC-C arm tended to identify as Muslim and the small number of women with no functional literacy (n = 3) were also in this arm (Table 2).

**Table 2:** Sociodemographic characteristics by randomization arm.

	Open-ended (IC-O) N = 40		Closed-ended (IC-C) N = 40		Total N = 80	
Characteristic	n	%	n	%	n	%
**Age (in Years)**						
18–24	17	(42.5)	15	(38.5)	32	(40.5)
25–29	12	(30.0)	11	(28.2)	23	(29.1)
30–36	11	(27.5)	13	(33.3)	24	(30.4)
Mean (SD)	26.3	(4.6)	27.2	(4.8)	26.7	(4.7)
Median (Range)	26	(18–35)	26	(19–35)	26	(18–35)
**Primary Education completed?**						
no school or primary school	25	(62.5)	28	(70.0)	53	(66.3)
Higher than Primary school	15	(37.5)	12	(30.0)	27	(33.8)
**Religion**						
Moslem	4	(10.0)	11	(27.5)	15	(18.8)
Christian	36	(90.0)	29	(72.5)	65	(81.3)
**Marital Status**						
Never married	14	(35.0)	14	(35.0)	28	(35.0)
Married or Cohabitating	23	(57.5)	19	(47.5)	42	(52.5)
Divorced, Separated or Widowed	3	(7.5)	7	(17.5)	10	(12.5)
**Functional Literacy**						
No	0	(0.0)	3	(7.5)	3	(3.8)
Yes	40	(100)	37	(92.5)	77	(96.3)
**Know someone who has ever participated in a SRH clinical trial**						
No	31	(77.5)	32	(80.0)	63	(78.8)
Yes	9	(22.5)	8	(20.0)	17	(21.3)
Total	40		40		80	
**Know someone one who is suspected to have died from AIDS**						
No	4	(10.0)	3	(7.5)	7	(8.8)
Yes	36	(90.0)	37	(92.5)	73	(91.3)

In the IC-C arm overall comprehension scores ranged from 23 to 39 and perceived ease of comprehension from 18 to 39 (25^th^–75^th^ percentile ranges 30 to 35 and 32 to 39, respectively). In the IC-O arm overall comprehension ranged from 3 to 38 and perceived ease from 0 to 39 (25^th^ to 75^th^ percentile ranges 19 to 31 and 30 to 39, respectively). Women in the IC-C arm had significantly higher overall comprehension scores than in the IC-O arm (p<0.01) with a mean score difference of 8.5 (95% CI 5.5, 11.5). This trend is reflected in the component comprehension scores with the exception of voluntariness, where the difference in score was minimal and slightly favored the open-ended assessment (Table 3).

**Table 3:** Comprehension scores and perceived ease of understanding of informed consent content, overall and by components, by randomization arm (mean, standard deviation).

		Closed-Ended Arm (IC-C)		Open-Ended Arm (IC-O)	
Components	# of Items	Comprehension	Perceived ease	Comprehension	Perceived ease
**Purpose of the research**	6	5.0 (0.8)	4.9 (1.4)	3.8 (1.5)	4.5 (1.4)
**Study procedures**	9	7.2 (1.4)	8.3 (1.3)	5.4 (2.8)	8.0 (1.8)
**Possible risks**	8	7.0 (1.2)	6.5 (1.9)	5.4 (2.3)	6.2 (2.5)
**Possible benefits**	8	6.6 (0.6)	7.4 (1.2)	3.6 (2.3)	7.2 (1.6)
**Confidentiality**	3	2.4 (0.6)	2.7 (0.6)	1.9 (1.1)	2.9 (0.5)
**Contacts for questions about research and rights**	2	1.9 (0.3)	1.9 (0.4)	1.3 (0.8)	1.9 (0.4)
**Voluntariness**	3	2.2 (0.9)	2.8 (0.5)	2.4 (0.7)	2.8 (0.6)
**Overall**	39	32.4 (3.6)	34.4 (5.8)	23.8 (8.8)	33.4 (7.4)

Overall perceived ease of comprehension scores (IC-SP) were similar between arms, with a mean difference of 0.95 (95% CI −2.0, 3.9; p = 0.526). Within arms, IC-SP scores were significantly higher when compared to the IC-O scores (mean difference 9.6, 95% CI: 6.6 to 12.6, p<0.01) and the IC-C scores, (mean difference 2.0, 95% CI: 0.35 to 3.6, p = 0.018).

Most participants expressed willingness to participate in the hypothetical trial described in the mock informed consent, regardless of randomization arm. Four women in the IC-O arm were unwilling to participate (IC-O scores 22–34, IC-SP scores 29–36), and three in the IC-C arm (IC-C scores 23–39, IC-SP scores 18–36).

## Discussion

Our findings confirm those from other assessments indicating that comprehension may appear higher when closed-ended measures are used compared to open-ended measures [19]. This held overall as well as by comprehension point, with the exception of comprehension of the voluntariness of participation which was similar in both arms. Comprehension of the right to refuse participation and to withdraw from participation without suffering negative consequences tends to be lower in developing country settings [28]. In many settings this may be less an issue of poor comprehension and instead reflect very real differences between acknowledged rights (e.g., to access health care services regardless of research participation) and the realities of exercising those rights (e.g., if in fact health care services provided as part of clinical trials are inherently superior to what is available outside a trial).

Overall perceived ease of comprehension was high relative to both IC-C and IC-O comprehension scores, confirming findings from at least one other study [15] and suggesting that it is not adequate to simply ask participants if they had any questions, were confused about, or understood each of the elements in the informed consent document. Some form of probing about the specific content, whether in an open-ended or closed-ended way, appears needed to bring a lack of comprehension to the surface and ensure an opportunity to address it.

Comparatively, the lowest score was observed in the comprehension assessment for possible benefits in the IC-O arm. Other components showed closer levels among comprehension assessment tools across the two arms. This could reflect the multiple points of comprehension assessed with a single open ended question and probes for the benefits component, while in the IC-C and IC-SP each separate question served as a reminder of each point of comprehension as well as the number of points to remember. Thus, the low IC-O score may suggest that the open ended line of questioning is less effective for questions requiring recall of multiple points, though it is not possible to disentangle the potential biases of the measurement approach. Future studies should attempt to clarify this point through a more strategic use of probes. An appropriate compromise approach could also entail use of multiple choice recall responses.

It is difficult to draw hard conclusions from our findings with regard to the best method for assessing informed consent comprehension, due to the lack of a “gold standard” measure. One interpretation would be that perceived difficulty of the information is least accurate, closed-ended measures slightly more accurate, and open-ended assessment most accurate for identifying lack of comprehension. However, it is possible that the open-ended approach used here may have overestimated poor comprehension on at least some items, for example if cultural or gender norms cause participants to be reticent in their responses.

Results from a generic open ended approach such as used in this study could be viewed as an overestimate of poor comprehension (or an underestimate of comprehension) and used as a conservative measure. This paired with evidence of comprehension of key points or components may be adequate for guiding and documenting the informed consent process. However, it is more challenging to determine a threshold for when a participant should not be enrolled because her level of comprehension of the study is inadequate for informed consent. In our study, the mean score in the open ended assessment indicated an average 61% comprehension. Could we consider this an acceptable level? Higher variability in the scores was also observed in the open ended group indicating less reliability and a greater number of participants with much lower scores who could potentially be turned down for enrollment due to lack of documented comprehension.

Our data are limited with respect to the study of willingness to participate. We found high levels of willingness to participate, but due to the small sample size we could not determine whether willingness may be associated with comprehension. Given the wide range of comprehension scores among the few participants unwilling to participate in the potential trial, we hypothesize that stated willingness to participate may not be associated with comprehension. For example, social desirability may have influenced these responses. These constraints combined with the known limitations of willingness to participate as a proxy for actual participation [29] point toward the need for additional research on comprehension in the context of actual clinical trials.

With these caveats in mind, our findings highlight the importance of comprehension assessments in complex clinical trials that go beyond asking participants if they have any questions. There is a need for continued exploration of objective measures of comprehension in international clinical research settings, so that points in need of clarification can be efficiently and effectively identified and addressed. Such measures would reduce burdens on both staff and participants that result from well-intentioned but potentially unnecessary time spent explaining in unwarranted detail things already understood. These findings highlight the need for identifying better measures of comprehension as a key step in improving the informed consent process overall.
